# Cancer Cell Membrane Decorated Silica Nanoparticle Loaded with miR495 and Doxorubicin to Overcome Drug Resistance for Effective Lung Cancer Therapy

**DOI:** 10.1186/s11671-019-3143-3

**Published:** 2019-11-08

**Authors:** Jinyuan He, Chulian Gong, Jie Qin, Mingan Li, Shaohong Huang

**Affiliations:** 1 Department of Cardiothoracic Surgery, The Third Affiliated Hospital of Sun Yat-sen University, Guangzhou, 510630 China; 2 Department of Anesthesiology, The Third Affiliated Hospital of Sun Yat-sen University, Guangzhou, 510630 China; 3 Department of Radiology, The Third Affiliated Hospital of Sun Yat-sen University, Guangzhou, 510630 China; 4 Department of Interventional Radiology, The Third Affiliated Hospital of Sun Yat-sen University, Guangzhou, 510630 China

**Keywords:** Cancer cell membrane, Silica nanoparticle, miR495, Doxorubicin, Drug resistance, Lung cancer therapy

## Abstract

Current cancer therapy usually succumbs to many extracellular and intracellular barriers, among which untargeted distribution and multidrug resistance (MDR) are two important difficulties responsible for poor outcome of many drug delivery systems (DDS). Here, in our study, the dilemma was addressed by developing a cancer cell membrane (CCM)-coated silica (SLI) nanoparticles to co-deliver miR495 with doxorubicin (DOX) for effective therapy of lung cancer (CCM/SLI/R-D). The homologous CCM from MDR lung cancer cells (A549/DOX) was supposed to increase the tumor-homing property of the DDS to bypass the extracellular barriers. Moreover, the MDR of cancer cells were conquered through downregulation of P-glycoprotein (P-gp) expression using miR495. It was proved that miR495 could significantly decrease the expression of P-gp which elevated intracellular drug accumulation in A549/DOX. The in vitro and in vivo results exhibited that CCM/SLI/R-D showed a greatly enhanced therapeutic effect on A549/DOX, which was superior than applying miR495 or DOX alone. The preferable effect of CCM/SLI/R-D on conquering the MDR in lung cancer provides a novel alternative for effective chemotherapy of MDR cancers.

## Introduction

Recent studies are offering accumulating evidences to the positive correlations between multidrug resistance (MDR) and the failure of chemotherapy [1, 2]. One of the most widely recognized mechanisms for MDR is the of ATP-binding cassette (ABC) transporter, among which P-glycoprotein (P-gp) is the mostly studied one [1, 3]. It was found that P-gp could effectively pump intracellular chemotherapeutics out of cells, leading to the reduced intracellular drug accumulation, as a result followed by the reduced efficacy [4, 5]. The inhibition of P-gp has shown beneficial effects on overcoming MDR, which might be a potential target to combat MDR.

MicroRNAs (miRNA) are a natural occurring type of non-coding RNA. However, miRNA shows important roles in the regulation of cellular transfection and protein expression [6]. As a result, it was reported that the expression of P-gp is affected by various miRNAs which depends on the tumor types [7]. Previous study has revealed that miR495 could effectively downregulate the P-gp expression in both MDR ovarian and gastric cancer cells [8]. Here, in this study, miR495 was employed to further explore its role in the regulation of P-gp in MDR lung cancer cells.

Due to the incomparable advantages, such as greatly reduced side effects along with increased bioavailability, drug delivery systems (DDS) have grown over the past decades and been recognized as the alternative to free drugs in drug delivery, especially in cancer therapy [9–12]. As a result, the development of multifunctional DDS capable of conquering the complicated extracellular and intracellular barriers of cancer therapy while at the same time suitable for the loading of various kinds of drugs is becoming the research focus [13–16]. Silica (SLI) nanoparticles, as one of the most widely adopted candidates, have versatile virtues such as easy preparation, high drug-loading capacity, and good biocompatibility and are preferable nanocarriers. Naturally, SLI has been used by many DDS as the nanocarrier for achieving satisfying efficacy [17, 18].

However, preclinical studies have revealed that targeted delivery of DDS is also vital for successful cancer therapy. To achieve better targeting effect, the most commonly adopted approach is to modify targeting ligands on the surface of DDS, which can bind to corresponding receptors on the surface of cancer cells [16, 19, 20]. From small molecules (molecular weight under 1000 Da) to monoclonal antibodies (molecular weight over 10 kDa), these targeting ligands have been reported to be successfully applied to DDS [21–23]. However, due to the ectogenic nature of some ligands or other reasons, adverse effects such as immunoreaction and cytotoxicity occurred. In recent years, cellular plasma membranes are becoming another promising material not only to modify the surface of nanoparticles but also to serve as a biocompatible targeting component. On the basis of the interaction between homologous cancer cell membrane (CCM) with cancer cells, the CCM has been shown to greatly increase the tumor-homing capability of DDS [24, 25].

In order to combine the tumor-homing property of CCM and P-gp targeting miR495 in one DDS for cocktail therapy of lung cancer (selected as a model cancer in our study), positively charged amine SLI was firstly fabricated and preloaded with doxorubicin (DOX). The DOX-loaded amine SLI was subsequently loaded with miR495 to form the co-delivery core (SLI/R-D). Finally, the SLI/R-D was decorated with negatively charged CCM (acquired from A549/DOX cells) to prepare a co-delivery and tumor-targeting DDS (CCM/SLI/R-D). It was expected that CCM can specifically guide the CCM/SLI/R-D to the homogenous A549/DOX cell to enhance its tumor-targeting effect and increase intracellular uptake. Meanwhile, the released miR495 can overcome the MDR of A549/DOX and achieve synergetic anticancer effect with DOX.

## Materials and Methods

### Materials

Methyl thiazolyl tetrazolium (MTT), *N*-(2-aminoethyl)-3-aminopropyltrimethoxysilane (AEAPS), tetraethyl orthosilicate (TEOS), doxorubicin (DOX), and Triton X-100 were obtained from Sigma-Aldrich (St. Louis, MO, USA). The miR495 was provided by Cell Biolabs Inc. (San Diego, CA, USA). Other chemicals and reagents were obtained from Aladdin Co., Ltd (Shanghai, China) and of analytical pure.

### Cell Culture and Animal Model

A549 (human lung carcinoma), A549/DOX (DOX-resistant cell line), and NIH3T3 (mouse embryonic fibroblast) cell lines were cultured in DMEM supplemented with 10% (v/v) fetal bovine serum (FBS), penicillin (100 U/mL), and streptomycin (100 U/mL) in a 37 °C constant temperature incubator containing 5% CO_2_. The A549/DOX (DOX-resistant cell line) was established by incubating A549 cells with gradually increased concentration of DOX as reported [26]. All cell lines were cultured in standard protocol as reported previously [27]. Male Balb/c nude mice (∼ 20 g) were obtained from the Institute of Model Animal, Wuhan University (Wuhan, China), and were raised with standard protocols. The A549/DOX tumor xenograft model was established based on previous article [28]. All animal-related experiments were approved by institutional Ethics Committee of the Third Affiliated Hospital of Sun Yet-sen University.

### Multicellular Tumor Spheroid Model

The multicellular tumor spheroid (MCTS) was established based on previous report [29]. In brief, a 96-well plate (Corning, USA) was covered with autoclaved agarose solution to create a gel pad. Afterwards, mixed A549/DOX and NIH3T3 cells (1:1) were seeded into the plate and incubated to form MCTS. The formation of MCTS was monitored by optical microscope (CX 23, Olympus, Japan).

### Preparation of CCM/SLI/R-D

The fabrication of amine SLI was carried out in a water-in-oil microemulsion based on previous report [30].Briefly, a DOX-contained water-in-oil microemulsion was fabricated at room temperature. Afterwards, AEAPS, TEOS, and NH_4_OH were successively added to trigger the reaction. After reacting for 24 h, the DOX-loaded amine SLI was precipitated using excess amount of ethanol and collected using centrifugation (3000 rpm, 10 min).

The miR495 was dissolved in HEPES buffer and dropwise added into the aqueous solution of DOX-loaded amine SLI at various weight/weight (w/w) ratios with vortex to obtain SLI/R-D binary complexes [31].

The isolation of CCM from A549/DOX cells was carried out based on previous report [32]. In summary, the A549/DOX cells were collected and concentrated using centrifugation. Afterwards, the cells were dispersed in extracting buffer and further centrifuged (10,000*g*, 10 min), followed by a second ultracentrifugation (100,000*g*, 60 min) to finally obtain the CCM. All procedures were performed at 4 °C. The protein concentration of CCM was quantified using a BCA kit (Beyotime, Shanghai, China).

The coating of CCM onto SLI/R-D used similar protocol as miR495 binding. In summary, different volumes of CCM solution were added to SLI/R-D (1 mg/mL) under vortex. Finally, the mixture was treated with probetype sonication (100 W, 5 min) and then was centrifuged (10,000*g*, 10 min) to obtain the CCM/SLI/R-D.

### Characterization

The size distribution and zeta potential of CCM/SLI/R-D were assessed by a Zetasizer (ZS90, Malvern, UK). In addition, the transmission electron microscope (TEM, JEM1230, JEOL, Japan) was applied to observe the morphology of nanocarriers.

The binding ability of SLI to miR495 was studied by gel retardation assay with naked miR495 as a control. The SLI/R-D formulated at various w/w ratios (0.2–25, SLI to miR495) was loaded on 2% agarose gel containing Goldview (Solarbio Science & Technology Co., Ltd., Beijing) and electrophoresed in 0.5× Tris-Borate-EDTA buffer (90 V, 60 min). The visualization of miR495 was performed using Gel-Pro analyzer (Genegenius, Syngene, UK).

The lysis buffer (Beyotime, Shanghai) was used to exact the total protein from CCM, after which the BCA kit was used for concentration quantification. Then, the samples were transferred onto poly(vinylidene fluoride) (PVDF) membrane. Finally, the membrane was stained with corresponding first antibodies (Abcam, USA) and second antibodies (Abcam, USA). The densitometer (E-Gel Imager, Thermo-Fisher, USA) was used for visualization.

The drug loading content (DLC) of CCM/SLI/R-D was determined by emerging the as-prepared nanocarriers in methanol for 48 h. The samples were centrifuged (10,000 rpm, 30 min), and the supernatants were collected for the determination of DOX by high-performance liquid chromatography (HPLC) [33]. The miR495 loading was determined by UV absorbance at 260 nm (UV5Nano, METTLER TOLEDO, Switzerland).

The changes of particle size of CCM/SLI/R-D in PBS and mouse plasma were recorded at each time point within 48 h. The release profile of DOX from CCM/SLI/R-D was investigated according to previous report [34].

### Intracellular Transfection

A549/DOX cells were seeded in 6-well plates for 24 h and then cultured with CCM/SLI/miR495 (miR495 concentrations, 1–25 ng/mL) for 48 h. Afterwards, cells were detached, harvested, and subjected to western blot analysis of P-gp expression.

The intracellular concentration of drug was determined according to previous report [23]. In brief, after treated with CCM/SLI/miR495 for different time intervals, the A549/DOX cells were incubated with DOX. At predetermined time intervals (4 and 8 h), cells were detached, collected, and dispersed in 5 mL of DOX extracting solution (ethanol 0.6 M HCl, 1:1, v/v), followed by ultrasonication at 400 W in ice bath for 40 times. The mixture was left at 4 °C for 24 h and centrifuged at 12,000 rpm (4 °C) for 10 min. The supernatant was collected and subjected to DOX content measurement as described above.

### Cell Viability

The cytotoxicity effect of drug-free nanocarriers (10–200 μg/mL) and CCM/SLI/R-D (DOX concentration, 2-50 μM; the miR495 concentration, 10–250 nM; the molar ratio between DOX and miR495 was fixed at 200) on A549/DOX cells for 48 h was determined by 3-(4,5-dimethylthiazol-2-yl)-2,5-diphenyltetrazolium bromide (MTT) assay. The level variations of apoptosis-related proteins were also determined using western blot assay.

MCTS with diameters of 300–400 μm was incubated with the medium containing different formulations (DOX concentration, 25 μM) at 37 °C for 5 days. An optical microscope was used to record the diameter changes of MCTS.

### In Vitro and In Vivo Targeting of CCM/SLI/R-D

The FAM-labeled siRNA was adopted to construct the DDS. A549/DOX cells were seeded in 6-well plates for 24 h. Then, the cells were incubated with excessive CCM for 2 h, and SLC/siRNA and CCM/SLC/siRNA were added. At the set time intervals, cells were collected and determined by flow cytometer (FCM, FC500MCL, Beckman Coulter) for quantification.

The Cy5 labeled miR495 was adopted to construct the DDS. Mice bearing A549/DOX tumor were i.v. injected with SLI/miR495 and CCM/SLI/miR495, and the distribution of miR495 was monitored at predetermined time intervals using real-time imaging system (ZEWTON 7.0, Vilber, France). After administration for 12 h, tumors and major organs were obtained from the sacrificed mice and imaged using the same system for analytical analysis.

### In Vivo Anticancer Assay

In vivo anticancer assay of CCM/SLI/R-D was assessed using A549/DOX tumor-bearing mice. In detail, mice were randomly divided into 5 groups (*n* = 6): (1) saline (as control), (2) free DOX, (3) CCM/SLI/DOX, (4) CCM/SLI/miR495, and (5) CCM/SLI/R-D. Afterwards, mice were intratumoral administered with the formulations at the dosage of 5 mg/kg DOX and 0.25 mg/kg miR495 for 7 times within 14 days. The tumor volumes and body weights of mice in each group were determined every 2 days.

## Results and Discussion

### Preparation of CCM/SLI/R-D

**Fig. 1:**
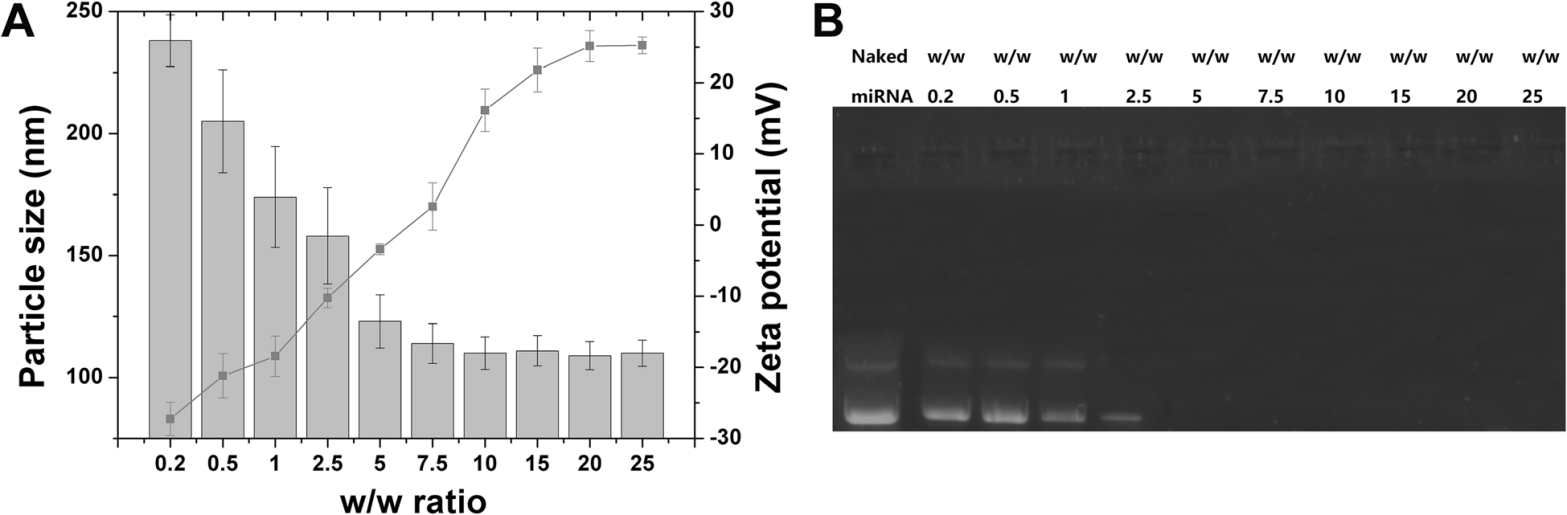
**a** Size and zeta potential changes of SLI/R-D at different w/w ratios. **b** The miRNA-binding assay of SLI/R-D binary complexes at various w/w ratios. Data were shown as mean ± SD (*n* = 3)

To achieve good drug-loading capacity and biocompatibility in one DDS, synchronous hydrolysis of TEOS and AEAPS in the water-in-oil microemulsion was used for the fabrication of SLI. DOX was pre-entrapped into the matrix of SLI during fabrication. As shown in Fig. 1a, the dynamic light scattering (DLS) result demonstrated that the DOX-loaded amine SLI had a diameter of ~ 100 nm. TEM image further revealed that the nanoparticles were spherical in shape with narrow distribution, which was in line with results obtained by DLS.

The DOX-loaded amine SLI had a surface potential of 26.83 mV, which was beneficial to serve as miR495 carrier. According to previous report [31], the assembly of miR495 and SLI to form binary complexes was driven by electrostatic interaction. The w/w SLI to miR495 in the binary complexes exerts significant impact on the final transfection efficiency. As displayed in Fig. 1a, due to the fact that miR495 is a negatively charged macromolecule, at low w/w ratio of SLI to miR495, the binary complexes have shown a negative surface charge with significantly increased particle size, which might be due to the adhesion of neighboring nanoparticles. However, with the increment of the w/w ratio, the surface charge of binary complexes gradually became positive with a stable particle size observed at around 100 nm. It was shown that when w/w ratio reached 20, both the particle size and surface potential of the binary complexes remained stable without significant change with the increase of w/w ratio.

The binding and protection capacities of the nanocarriers to miR495 are vital for gene delivery. The miR495-binding capability of SLI was assessed by gel retardation assay. As shown in Fig. 1b, naked miR495 showed no retardation while the adding of SLI significantly changed the behavior of miR495. It was observed that SLI showed growing miR495-binding capacity with the increment of w/w ratio, which achieved complete retardation of miR495 at the w/w ratio of 5.

To sum up, it was inferred that binary complexes at the w/w ratio of 20 with proper particle size and desirable surface charge, as well as effective miR495-binding and protection property, were the optimal formulation which was selected as the model to perform the following experiments.

**Fig. 2:**
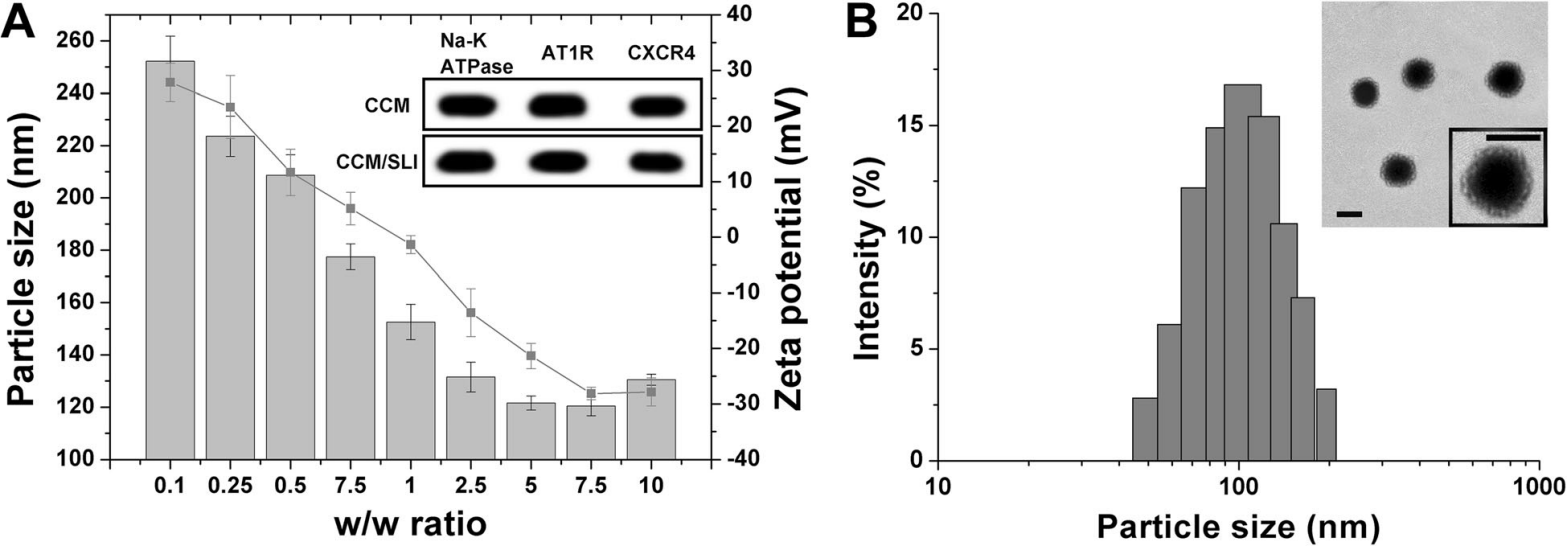
**a** Size and zeta potential changes of CCM SLC/R-D at different w/w ratios. Inserted image was a western bolt analysis of the three representative proteins in CCM and CCM/SLI/R-D. **b** The size distribution and TEM of CCM/SLI/R-D. Data were shown as mean ± SD (*n* = 3). Scale bars 100 nm

Then, we explored the optimal CCM protein ratio to the binary complexes by mixing CCM with binary complexes at different mass ratios (SLI to CCM protein, w/w). The optimal ratio was determined by the resulted particle size and surface charge under different conditions. As shown in Fig. 2a, the adding of CCM (negative charge) resulted in a significant fluctuation on the size of the product but continuous decrease on surface charge. The results indicated that CCM was successfully anchored on the surface of binary complexes. Most importantly, it was observed that both the particle size and surface charge of CCM/SLI/R-D reached a plateau at the mass ratio of 7.5 and that the additional CCM showed insignificant impact on surface charges and only mild increase on size. To be specific, the diameter of the nanoparticle reached 121.28 ± 3.36 nm and the zeta potential changed to − 28.04 ± 2.64 mV, which was similar to the surface charge of free CCM (− 27.95 ± 3.06 mV), suggesting that the decorating of CCM reached saturation under this condition. As a result, the CCM/SLI/R-D at the mass ratio of 7.5 was selected as the model formulation to perform the following experiments.

### Characterization of CCM/SLI/R-D

Previous reports have shown that proteins in CCM are capable of homing the homologous tumor cells when adopted to modify nanoparticles [35, 36]. Therefore, three membrane proteins (Na-K ATPase, AT1R, and CXCR4) were selected and their expression levels in CCM were compared with CCM/SLI/R-D. As shown in the inserted image of Fig. 2a, the amount of all three proteins in CCM showed similar intensity to that of CCM/SLI/R-D, which demonstrated that integrated proteins in CCM were inherited to CCM/SLI/R-D after coating and the lost or degradation was negligible during this process. This result also provided solid evidence to confirm the successful construction of CCM/SLI/R-D, which was beneficial for increased tumor homing of CCM/SLI/R-D.

The size distribution and morphology of CCM/SLI/R-D were also studied using DLS and TEM. As shown in Fig. 2b, DLS revealed that CCM/SLI/R-D was narrowly distributed at around 120 nm while TEM showed that CCM/SLI/R-D was characterized to be spherical core shell structure and a lipid bilayer could be clearly observed on the superficial layer.

The DLC of DOX in CM/SLI/R-D (determined by HPLC) could be as high as 17.96%, and the miR495 loading (determined by UV spectrophotometer) could be as high as 1.64%.

**Fig. 3:**
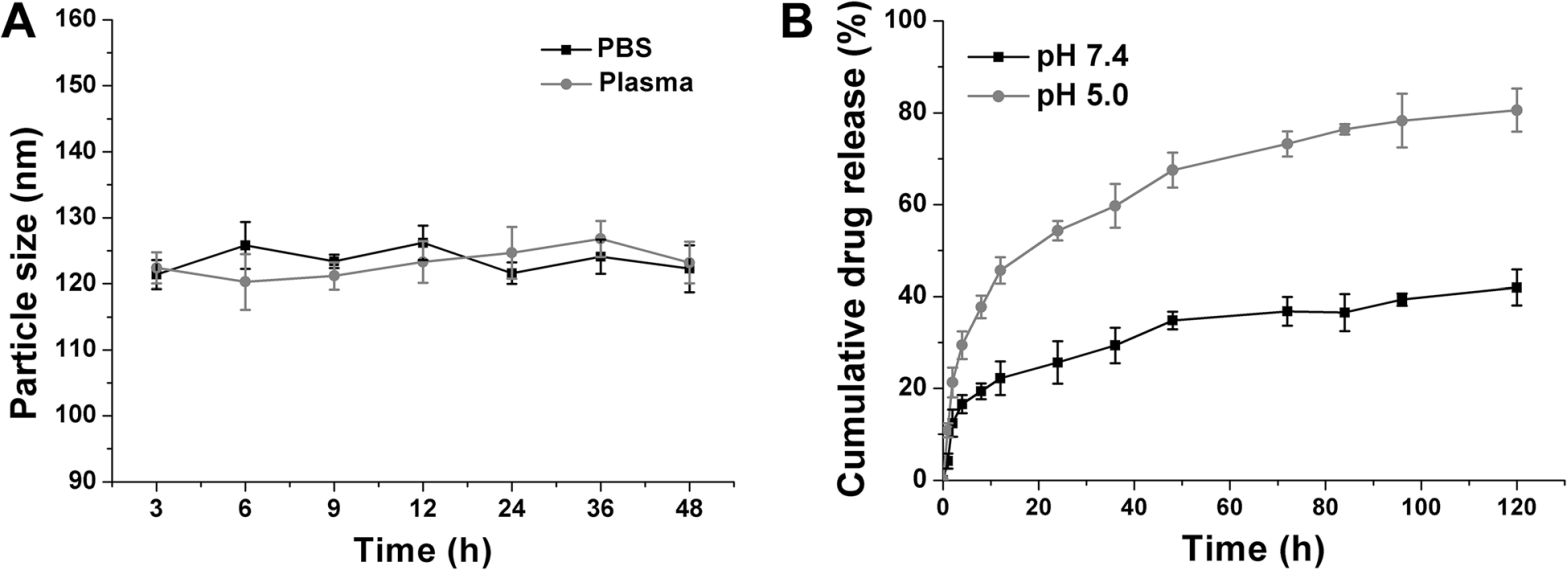
**a** Colloidal stability of LCC/R-A in PBS (pH 7.4) and mouse plasma at 37 °C for up to 48 h. **b** Drug release profiles of DOX from the LCC/R-A in release media under extracellular and intracellular condition of pHs (7.4 and 5.5). Data were shown as mean ± SD (*n* = 3)

Previous studies have concluded several preliminary requirements for safe delivery of drug molecules. First of all, the adopted DDS should be maintained stable without dramatic size variations under physiological environments, since particle size contributed critical significance to the in vivo fate of the system [10, 37]. As a result, the time-dependent stability of CCM/SLI/R-D was investigated. In order to determine the colloidal stability of CCM/SLI/R-D in physiological environments, the size changes of DDS in PBS (pH 7.4) and mouse plasma were recorded up to 48 h. As displayed in Fig. 3a, CM/SLN/Ce6 could effectively maintain its size during the whole test range without significant variations. Therefore, it was concluded that CCM/SLI/R-D was capable of maintaining stable in physiological condition. As shown in Fig. 3b, the CCM/SLI/R-D could preserve stability at extracellular condition (32.76% of drugs being release after 120 h of incubation), indicating that during the delivery process, the CCM/SLI/R-D could safety encapsulate the loaded drug molecule without inducing potential adverse effects. Most importantly, upon entering cells and being subjected to the acidic environment of cancer cells, the drugs were readily released (75.93%), which might ascribed to the high hydrogen concentration that weakens the combination of drug and carriers [6, 38].

### Intracellular Transfection

**Fig. 4:**
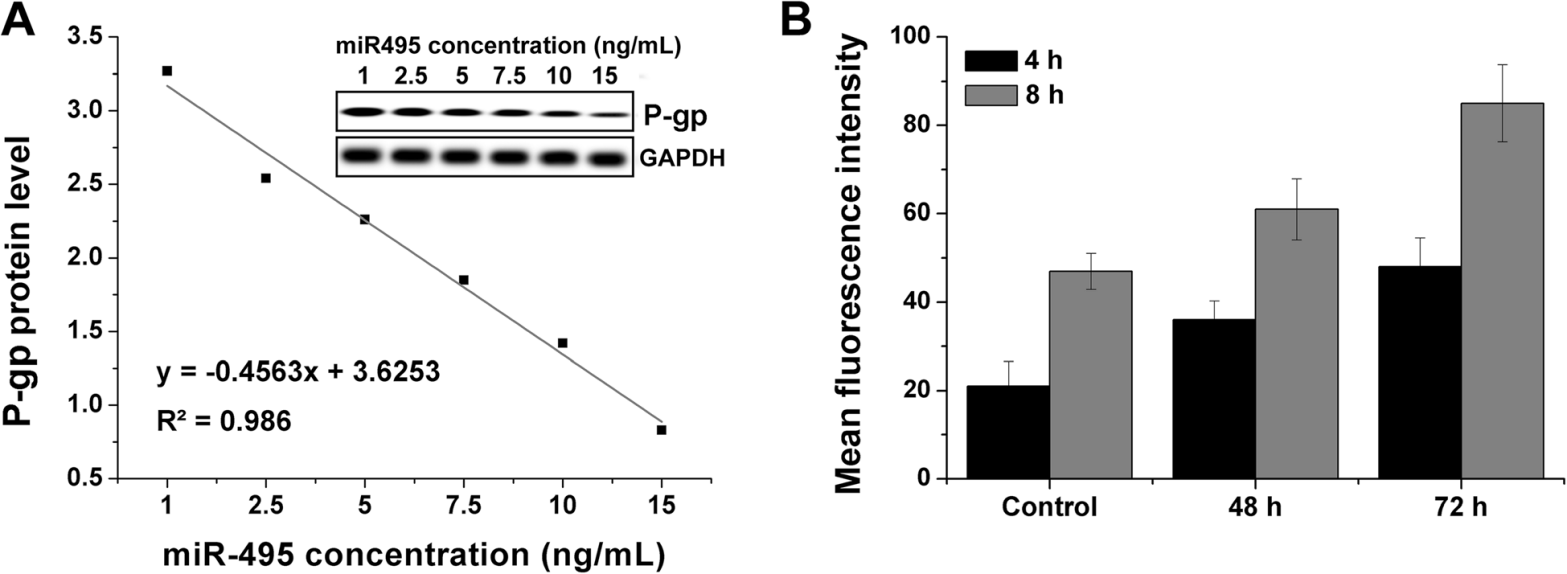
**a** The relation between miR495 concentration and the expression of P-gp proteins in A549/DOX cells after being treated with CCM/SLI/miR495 for 48 h. **b** The intracellular DOX accumulation in A549/DOX cells after being treated with LCC/miR495 for different time intervals. Data were shown as mean ± SD (*n* = 3)

**Fig. 5:**
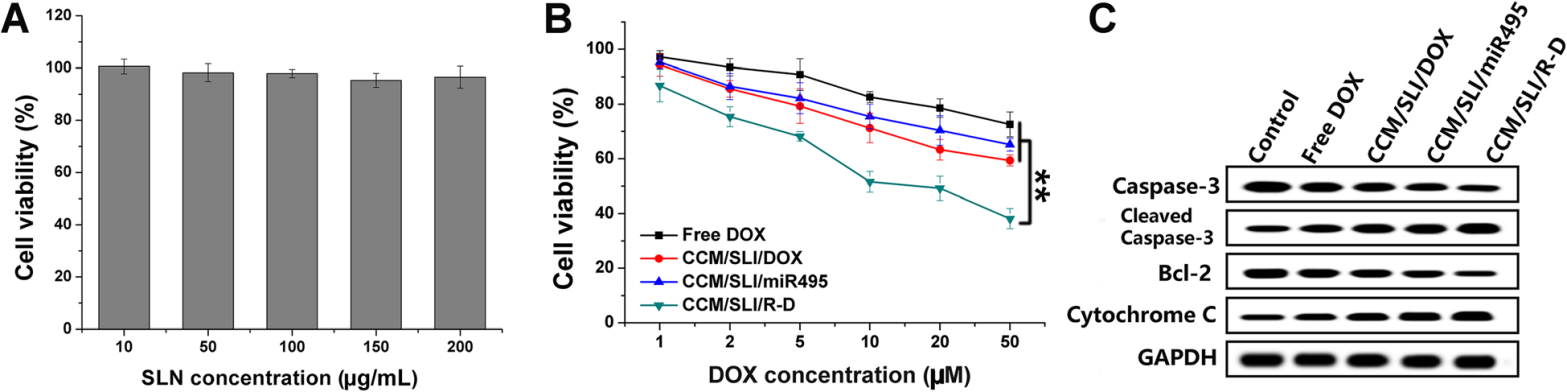
Viability of A549/DOX cells treated with drug free carrier (**a**) or drug containing (**b**) different formulations at different nanoparticle/drug concentrations for 48 h. **c** Western blot assays of the expression of caspase-3, cytochrome C, and bcl-2 proteins after different treatments. Data were shown as mean ± SD (*n* = 3). ^**^P < 0.01

In order to reveal the relation between the transfection of miR495 and the expression of P-gp, the A549/DOX cells were transfected with different concentrations of miR495. Afterwards, the P-gp expression level in these cells was determined using western blot assay. As shown in Fig. 4a, the expression of P-gp showed a negative relation to miR495 concentration, suggesting that the CCM/SLI could be applied as a useful tool for miR495 delivery, which is beneficial for CCM/SLI/R-D to reverse the MDR in A549/DOX cells. As a proof of concept, the effect of miR495 transfection on the variation of intracellular DOX accumulation was further studied. After being treated with CCM/SLC/siRNA for 48 or 72 h (Fig. 4b), the A549/DOX cells were treated with DOX for varied time intervals. As shown in Fig. 5d, with CCM/SLC/siRNA pretreatment for 48 h, the cellular fluorescence of DOX in A549/DOX cells increased 1.49-fold (4 h post-incubation) and 1.47-fold (8 h post-incubation), respectively. With CCM/SLC/siRNA pretreatment for 72 h, the intracellular DOX fluorescence in A549/DOX cells increased 1.63-fold (4 h post-incubation) and 1.85-fold (8 h post-incubation). As a result, it was concluded that CCM/SLC/siRNA could overcome the MDR in A549/DOX by increasing the intracellular DOX accumulation compared to untreated cells.

### In Vitro Anticancer Assay

The cytotoxicity of drug-free carrier (CCM/SLI) was studied to further reveal the biocompatibility of the DDS. As shown in Fig. 5a, after incubating with CCM/SLI for 48 h at the highest concentration of 200 μg/mL, the A549/DOX still showed more than 90% of viability, which suggested that CCM/SLI was biocompatible with insignificant toxic to cells.

Afterwards, the in vitro anticancer assay was evaluated. As shown in Fig. 5b, the anticancer effects of all formulations were positively related to drug concentrations. In detail, the viability of A549/DOX cells was still 72.6% at the highest DOX concentration (50 μM). The CCM/SLI/DOX and CCM/SLI/miR495 under the same condition achieved similar cell viability of which was 59.4% and 63.3%, respectively, which was higher than that of DOX. However, it was noted that the cell viability for CCM/SLI/R-D treatment was significantly decreased to 38.5%. In addition, the CI index in CCM/SLI/R-D was determined as 0.84, suggesting a strong synergistic effect of miR495 and DOX on the killing of A549/DOX cells.

In order to verify the conclusion again, the commonly adopted apoptosis regulation proteins (caspase-3, bcl-2, and cytochrome-3) were assessed in different formulations. As demonstrated in Fig. 5c, the amount of cleaved caspase-3 was the highest in CCM/SLI/R-D-treated cells and the bcl-2 (apoptosis suppressor) level was the lowest among all testing groups, which further confirming the preferable anticancer efficacy of CCM/SLI/R-D. In addition, the CCM/SLI/R-D showed much elevated expression of cytochrome-3, which suggested that the apoptosis in this group was related to mitochondria damage.

**Fig. 6:**
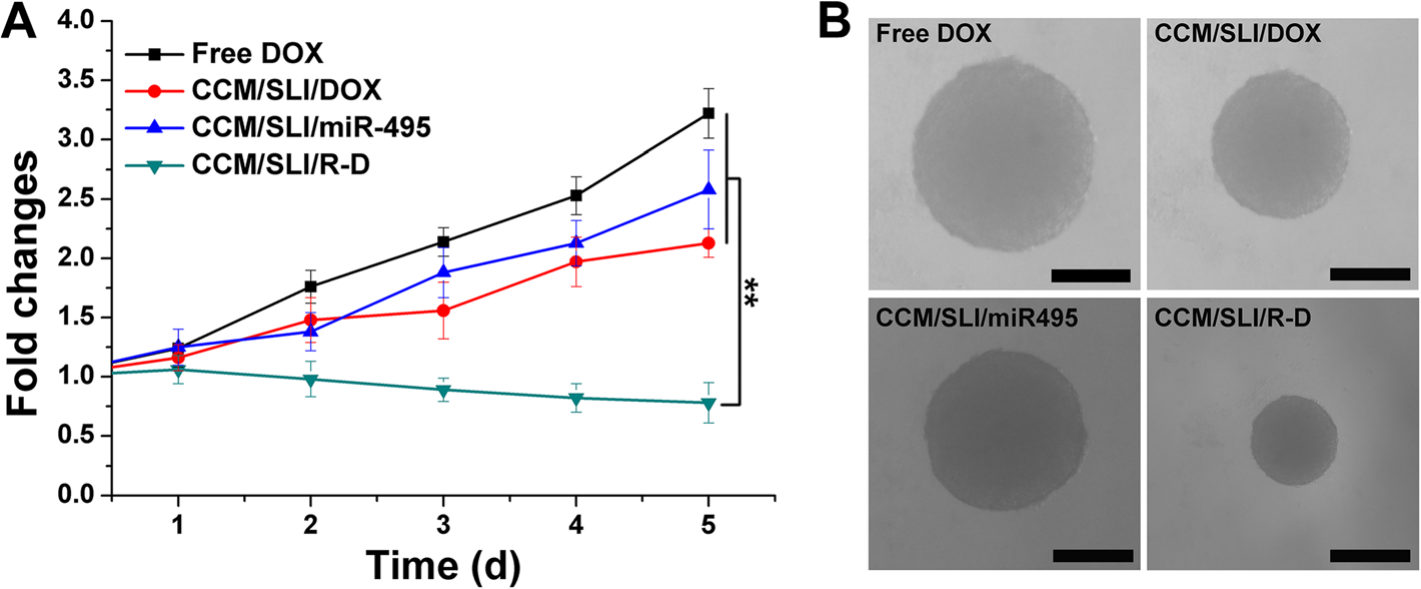
The volume changes (**a**) and corresponding optical images (**b**) of MCTS after different treatments. Data were shown as mean ± SD (*n* = 3). ^**^P < 0.01

The MCTS was adopted to mimic solid tumor and to assess the anticancer effect of different formulations. As shown in Fig. 6a, the MCTS volume in the free DOX group persistently grows in the whole experiment period, suggesting that the MDR in A549/DOX could significantly diminish the cytotoxicity of DOX. Single delivery systems (SLI/DOX and SLI/miR495) showed certain suppression effect with mildly decreased growth. Most importantly, the combination of miR495 and DOX in CCM/SLI/R-D showed greatly elevated efficacy with a negative volume growth observed at the end of the test. The optical image in Fig. 6b also reached similar conclusions.

### In Vitro and In Vivo Targeting

**Fig. 7:**
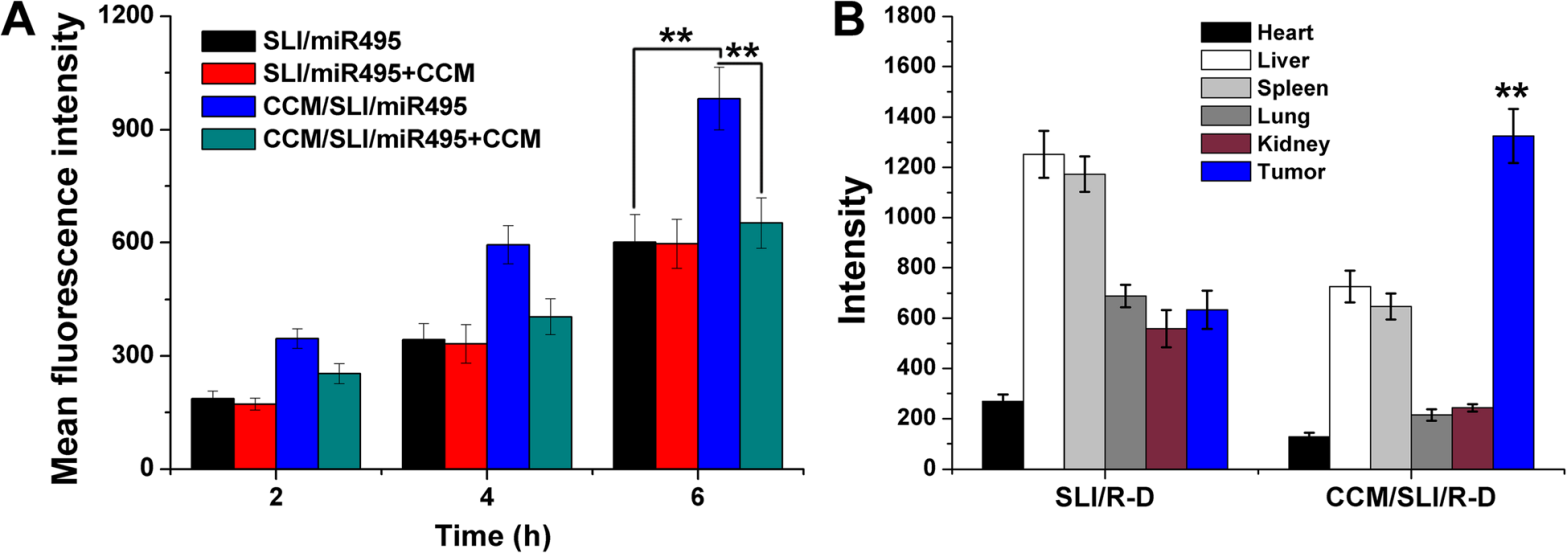
**a** Quantitative analysis of intracellular time-dependent uptake of different formulations in A549/DOX cells (pretreated with/without CCM). **b** Mean fluorescence intensity of dissected tumors and major organs of mice treated with SLI/R-A and CCM/SLI/R-A at 48 h post-injection. Data were expressed as mean ± SD (*n* = 3). ^**^P < 0.01

To reveal the possible reasons for the discriminative antitumor effect of various formulations, cellular uptake was assessed to investigate whether CCM modification could positively increase the internalization capacity of nanoparticle in A549/DOX cells since many studies have demonstrated that surface modification of CCM can enhance the tumor-targeting capacity of nanocarriers [39, 40]. As displayed in Fig. 7a, the intracellular fluorescence intensity increased in the CCM/SLI/miR49 and SLI/miR495 group as a function of time, suggesting the positive relation between time and cellular uptake in both nanoparticles. In addition, it was noted that higher fluorescence signals were observed in the CCM/SLI/miR495 group, which was 1.58-fold of that in the SLI/miR495 group at the time point of 6 h. To verify whether the uptake of CCM/SLI/miR495 was via the CCM-mediated endocytosis, the cells were incubated with excess CCM before the addition of nanocarriers. It was observed that the fluorescence intensity in the CCM/SLI/miR495 group continued to decrease, while the fluorescence intensity in the SLI/miR495 group remained almost the same level. These results demonstrated that CCM/SLI/miR495 was taken up by cells through CCM-related endocytosis.

The CCM from A549/DOX was expected to enhance the tumor-targeting capacity of CCM/SLI/R-D to isogenous A549/DOX cells to increase the accumulation of nanocarriers in tumor. To verify our concept, the distribution of SLI/R-D and CCM/SLI/R-D was monitored by a real-time imaging system. As shown in Fig. 7b, as expected, CCM/SLI/R-D showed more accumulation within the tumor as compare to SLI/R-D. In addition, it was concluded that SLI/R-D was distributed in almost all the organs (except the heart) with focus in the liver and spleen, which might be due to their poor tumor-homing capability. On the contrary, CCM modification could significantly alleviate liver capture to assist the enhanced homing of loaded cargo to tumor tissue.

### In Vivo Antitumor Efficacy

**Fig. 8:**
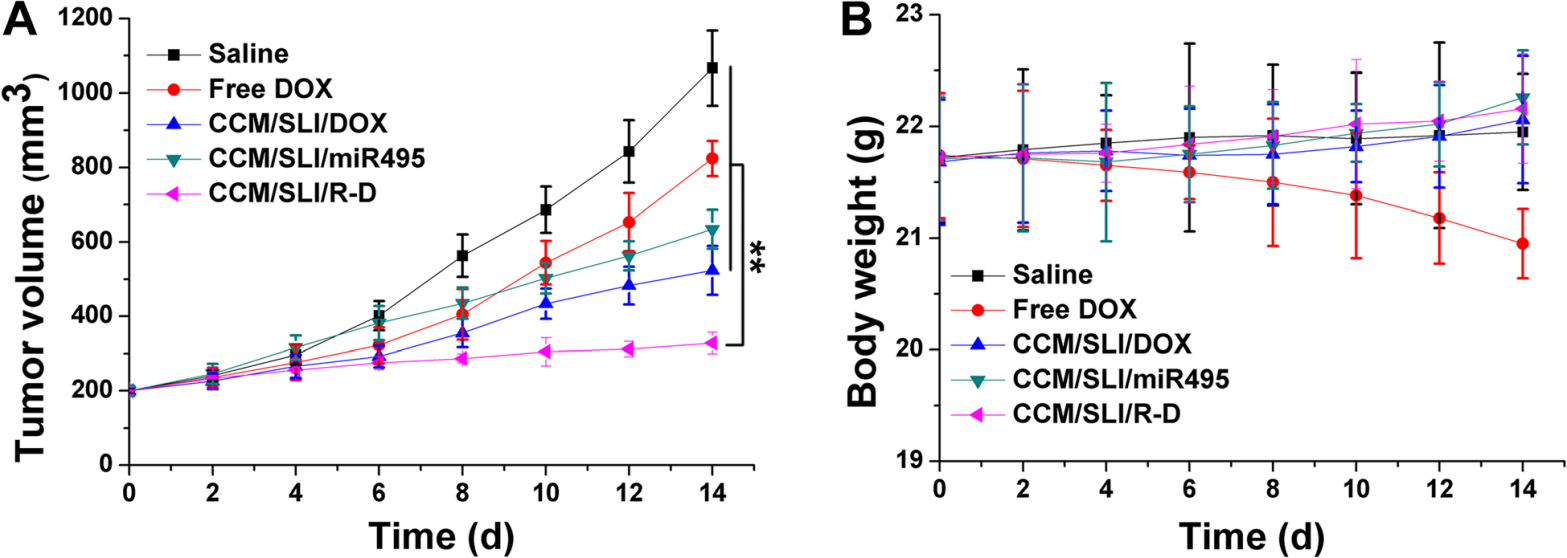
The tumor volume (**a**) and body weight (**b**) of tumor tissue analysis of A549/DOX tumor-bearing Balb/c nude mice after intratumoral administration of different formulations. Data were expressed as mean ± SD (*n* = 6). ^**^P < 0.01

In vivo antitumor efficacy of CCM/SLI/R-D was performed. After treatment with DOX or SLI/DOX, the tumor growth of mice was slowed down. However, CCM/SLI/R-D had the best antitumor efficacy and resulted in an obviously reduced tumor volume of 303 ± 25 mm^3^. Moreover, body weight variation result of mice in different groups also displayed interesting results (Fig. 8). It showed there was no obvious decline of body weight in mice treated with CCM/SLI/R-D, suggesting that the tumor-targeting ability of CCM/SLI/R-D could enhance antitumor efficacy with reduced adverse effects. In contrast, the untargeted distribution of free DOX caused systematic toxicity to mice, which was reflected by the gradually decreased body weight as a function of time. In summary, the CCM/SLI/R-D was a superior tumor-homing DDS for lung tumor therapy.

## Conclusion

In summary, we successfully constructed a CCM-coated SLI nanoparticle as a DDS to co-delivery miR495 and DOX (CCM/SLI/R-D). The characterization demonstrated that CCM/SLI/R-D showed well distribution with a diameter of 120 nm, which showed high stability as well as pH-responsive drug release. Cellular experiments revealed that CCM/SLI/R-D could realize preferable miR495 delivery which achieved the significant downregulation of P-gp, which finally overcome the MDR in A549/DOX by increasing the intracellular DOX accumulation compared to untreated cells. The CCM/SLI/R-D showed promising tumor-homing capability. Most importantly, the synergetic effect of miR495 and DOX achieved much more potent anticancer effect to mono-delivery DDS or free DOX both in vitro and in vivo.

## Data Availability

The data and the analysis in the current work are available from the corresponding authors on reasonable request.

## Abbreviations

AEAPS: *N*-(2-Aminoethyl)-3-aminopropyltrimethoxysilane
CCM: Cancer cell membrane
DDS: Drug delivery systems
DLC: Drug loading content
DOX: Doxorubicin
HPLC: High-performance liquid chromatography
MCTS: Multicellular tumor spheroid
MDR: Multidrug resistance
miRNA: MicroRNAs
MTT: Methyl thiazolyl tetrazolium
P-gp: P-glycoprotein
SLI: Silica
TEOS: Tetraethyl orthosilicate
